# Eco-Friendly Copper Oxide Nanoparticles Incorporated Polyvinylidene Difluoride Electrospun Nanofibers as Wearable Piezoelectric Nanogenerator

**DOI:** 10.3390/polym18060699

**Published:** 2026-03-13

**Authors:** Amrutha Bindhu, Sathiyanathan Ponnan, Shamim Reza Mohammad, Riya Karmakar, Hongdoo Kim, Arvind Mukundan, Anand Prabu Arun

**Affiliations:** 1 Department of Chemistry, School of Advanced Sciences, Vellore Institute of Technology, Vellore 632014, India; amrutha.b2020@vitstudent.ac.in; 2 Department of Chemistry, Vel Tech Rangarajan Dr. Sagunthala R&D Institute of Science and Technology, Chennai 600062, India; sat.bni@gmail.com; 3 Department of Materials Science and Engineering, College of Engineering, Kyung Hee University, Yongin-si 17104, Gyeonggi-do, Republic of Korea; reza13@khu.ac.kr (S.R.M.); hdkim@khu.ac.kr (H.K.); 4 School of Engineering and Technology, Sanjivani University, Sanjivani Factory, Kopargaon 423603, India; karmakarriya345@gmail.com; 5 Department of Biomedical Imaging, Chennai Institute of Technology, Chennai 600069, India

**Keywords:** PVDF, CuO, nanofiber, piezoelectric nanogenerator, wearable sensors

## Abstract

This study focuses on enhancing the performance of piezoelectric nanogenerators (PENGs) fabricated by electrospinning (ES) of polyvinylidene fluoride (PVDF) infused with varying concentrations (0, 1, 3, 5, and 7 wt.-%) of copper oxide (CuO) nanoparticles. Structural changes and the *β*-phase proportion in nanofibers (NFs) were examined using XRD and FTIR-ATR. Surface morphology and roughness were characterized using FE-SEM and AFM, respectively. The water-repellent characteristics of the NFs were assessed through WCA measurements. Electrical output (voltage and current) was evaluated under mechanical pressure using a customized setup that applied 1.0 kgf at 1.0 Hz. The pristine PVDF-based PENG generated an output of 1.7 V and 0.53 μA, while the composite NF with 5 wt.-% CuO (5PCu) delivered a significantly enhanced output of 13.7 V and 1.6 μA. The 5PCu device was further tested for detecting human activities, including tapping, wrist movements, walking, and jumping, thereby demonstrating its potential for self-powered wearable electronics.

## 1. Introduction

Materials capable of harvesting energy from human physical activities (bending, walking, jumping) and biomechanical processes (respiration and heartbeat) have gained significant attention for powering portable and wearable sensors [1,2,3,4,5]. Wang et al. demonstrated the first piezoelectric nanogenerator (PENG) based on arrays of zinc oxide nanowires [6]. Since then, extensive research has focused on PENGs employing both organic and inorganic materials, notably polyvinylidene fluoride (PVDF) and its composites with inorganic fillers. The piezoelectric performance of PVDF is predominantly governed by its five distinct crystalline phases (*α*, *β*, *γ*, *δ*, and *ε*). Among them, the *β*-phase is highly electroactive and provides the strongest piezoelectric response due to the parallel alignment of dipoles in the CH_2_ and CF_2_ groups [7,8,9]. Achieving the *β*-phase requires different strategies, including mechanical stretching, high-temperature annealing, electrical poling, structural engineering, solution casting, electrospinning (ES), and compositing with nanofillers. ES is widely employed to promote the formation of *β*-phase in PVDF [10,11,12]. Polarization in a strong electric field has been shown to effectively enhance dipole alignment and piezoelectricity in PVDF [13]. However, this method is limited by the directional restriction of polarization, which reduces structural flexibility. However, combining ES and compositing strategies synergistically enhances *β*-phase without any supplementary post-processing steps, such as electrical poling and thermal treatment [14,15,16]. For instance, polyvinylidene fluoride (PVDF)/PZT nanofibers (NFs) prepared via electrospinning (ES) with varying PZT concentrations exhibited higher *β*-phase crystalline content compared to pure PVDF NFs, with 10% PZT doped PVDF yielding the highest output voltage of 79.7 mV under resistive load [17,18,19]. Similarly, the addition of fillers, such as BaTiO3 [20], CNT [21], graphene oxide [22], cobalt ferrite [23], and ZnO [24], has been reported to enhance the *β*-phase of PVDF by electrospinning. Despite the fact that these additives are highly effective at enhancing the formation of *β*-phases through interfacial polarization and dipole alignment during electrospinning, most of them have acute toxicities. Cytotoxicity, inhalation hazard, or environmental persistence is linked to lead-containing PZT, barium- and cobalt-based oxides, carbon nanomaterials, and metal nanoparticles, restricting their application as wearable piezoelectric materials. Despite excellent performance, the toxicity of the fillers used in PVDF composites raises serious environmental concerns, driving the current shift toward lead-free ceramic alternatives as eco-friendly piezoelectric materials.

To address these setbacks, an eco-friendly nanofiller is highly recommended. In this respect, Copper oxide (CuO) nanoparticles, owing to integrated interfacial, structural, and electrical charge-transport effects, are significantly involved in the nucleation of PVDF by providing sufficient charge-holding centers, thereby inducing surface polarization. This work demonstrates the fabrication of an eco-friendly copper oxide (CuO) NPs-doped PVDF membrane as PENG, showing excellent piezoelectric output performance of 13.7 V at 1.6 µA, which is comparable to many reported works listed in Table 1. This study enables the reliable manufacturing of highly efficient PENGs. The results are reported in detail in the following sections.

Since PVDF-based PENGs have received significant attention, most published methods for performance enhancement rely on conventional piezoelectric ceramic fillers, such as TiO_2_ [5], BaTiO_3_ [20], ZnO [24], and PZT [19]. The final result enhancement is mainly due to the fundamental piezoelectric effect of the filler. Alternatively, the inclusion of non-conventional oxide fillers with low inherent piezoelectricity, especially CuO, is relatively unresolved in PENG technologies. Our previous study described a triboelectric nanogenerator (TENG) device comprising a CuO-PVDF/PU bilayer [25], demonstrating performance enhancements enabled by contact electrification between two tribo-materials. That research focused primarily on improving triboelectric output and the capabilities of flexible sensors. The fundamental relation between nanoparticle loading and structural optimization, specifically the reason for performance degradation at greater filler concentrations, is still not quantitatively defined. In this regard, the current study presents a comprehensive structure–property–performance study of CuO-doped PVDF nanofibers in a PENG configuration. This study identifies an optimal loading (5 wt.-%) and analyzes competing structural effects at higher concentrations by systematically linking CuO concentration to crystalline phase composition, microstructural evolution, and electrical performance. Thus, this work closes a significant gap in the logical optimization of PVDF-based PENG systems built by nanoparticles utilizing non-conventional fillers.

## 2. Materials and Methods

### 2.1. Materials

PVDF powder (MW ~370,000) was bought from Solvay, Seoul, Republic of Korea. Acetone and dimethylformamide (DMF) solvents were supplied by Merck, Mumbai, India. Ni-Cu-infused electrodes were supplied by Solueta Co., Ltd., Gyeonggi-do, Republic of Korea. Sodium hydroxide (98%) and Copper sulfate pentahydrate (CuSO_4_·5H_2_O) were purchased from Sigma Aldrich, Bangalore, India. All materials were used without any further purification.

### 2.2. Synthesis of CuO NPs

**Figure 1:**
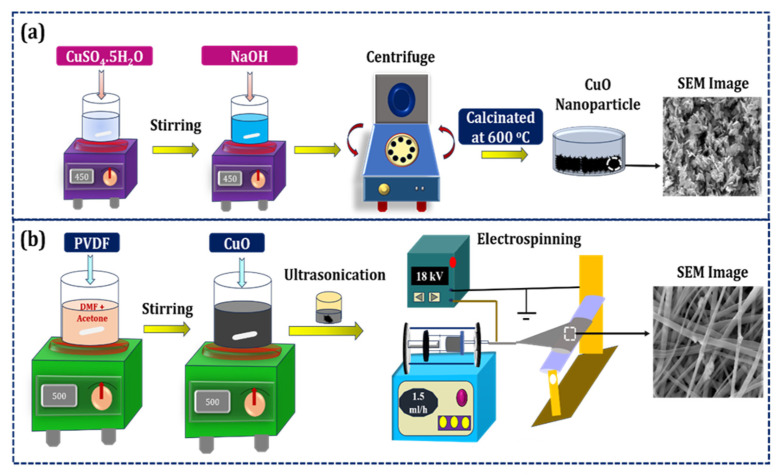
(**a**) Visual representation of CuO NP preparation; (**b**) Production of CuO-doped PVDF-based ES NF.

Figure 1a shows the step-wise synthesis of CuO NPs using the chemical co-precipitation method. Initially, CuSO_4_·5H_2_O precursor material dissolved in DI water was continuously stirred for 2 h, followed by drop-wise addition of NaOH (0.1 M) solution to the precursor solution till it reached pH 14. After the completion of precipitate formation, the resultant solution mixture was centrifuged and washed with DI water. Lastly, the precipitate was calcinated at 500 °C for 5 h [26].

### 2.3. Fabrication of CuO-PVDF ES NF

**Figure 2:**
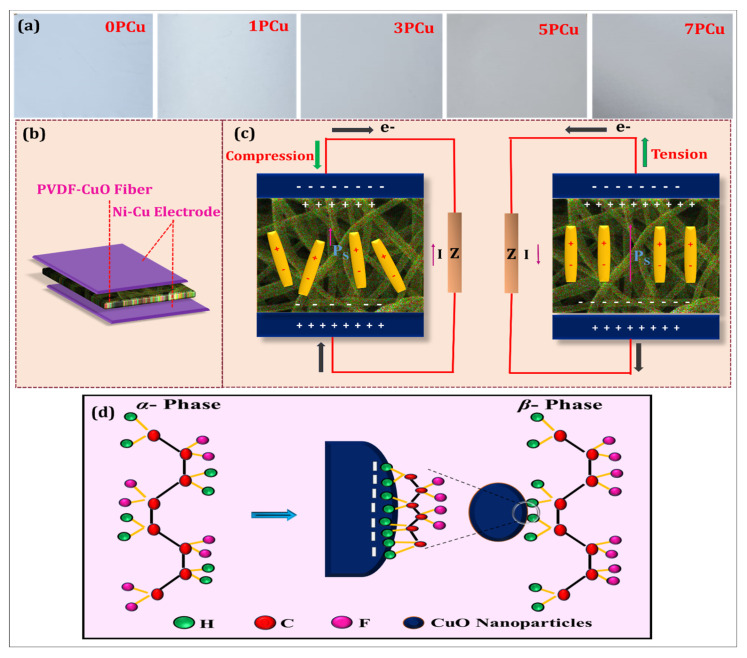
(**a**) Images of the fabricated neat and composite ES NFs; (**b**) Illustration of the layer orientation; (**c**) Charge-generating mechanism in the PENG device; (**d**) Schematic representation of the ion-dipole interaction of the PVDF chains with CuO NP.

Neat PVDF and CuO-PVDF composite NF were fabricated using the ES technique, as graphically depicted in Figure 1b. For preparing the ES solution, 1.2 g of PVDF powder (12 wt.-%) was dissolved in an acetone/DMF mixture (2:3) and stirred continuously for 3 h at 500 rpm and ambient temperature. Then, four different weight % of CuO NPs (1, 3, 5, and 7 wt.-%) were added to the PVDF solution and stirred continuously for 10 h, followed by 3 h of ultrasonication to homogenize the solution. The as-prepared solution was loaded into a 10 mL syringe fitted with a 21 G needle for ES. A Teflon sheet was used for collecting the NF, and the spinning conditions were set at applied voltage = 18 kV, distance from needle to collector = 10 cm, flow rate = 1.0 mL/h, and spinning time = 5 h. The photographic images of neat PVDF (0PCu) and CuO-PVDF (1PCu, 3PCu, 5PCu, and 7PCu) composite NFs are shown in Figure 2a.

### 2.4. Fabrication and Working Principle of the PENG Device

Figure 2b depicts the PENG device configuration, in which Ni-Cu-coated conductive materials (2 × 2 cm^2^) function as top as well as bottom electrodes. The size of the ES NC NF placed between the two electrodes is kept larger than the electrodes to prevent short-circuiting. Giving a sudden external mechanical force to the developed sensor creates a pulse-type electric pattern. The presence of vertical stress changes the arrangement of dipoles within the composite, resulting in the production of piezoelectric voltage as illustrated in Figure 2c. A positive voltage generated by the nanogenerator matches the initial positive peak. Whenever the vertical pressure is removed, the dipole orientation inside the NC is distorted, and simultaneously, the potential between the electrodes decreases. As a result, the generated charges are carried backward in the opposite direction, generating a negative peak [27,28,29,30].

### 2.5. Polarization Mechanism

Based on FTIR and XRD results of the composite fibers, the addition of CuO NP to the surface of PVDF enhances the strengthening of the polar *β*-crystalline phase in PVDF. Then, the mechanism of interaction between CuO and the PVDF matrix should be understood. The NP synthesis step involved using NaOH to maintain the solution at pH 14. Therefore, the zeta potential of the NP depends on the pH of the precursor solution. The NP produced has a negative surface, as evidenced by the zeta potential at pH 14. According to earlier research, CuO NP dissolved in polar solvents (DMF) typically exhibit a negative surface charge due to the presence of hydroxyl groups on their surface [31]. The use of nanoparticles may aid dipole orientation during electrospinning, leading to increased charge generation. This behavior could be explained by possible surface polarization effects between the nanoparticles and the polymer matrix. Since no direct interfacial characterization techniques, such as XPS or TEM, were used in this study, the observed electrical and structural patterns support this interpretation. FTIR study verifies an increase in *β*-phase, but does not directly investigate interfacial interactions.

When they interact, the negatively charged NP surface serves as a substrate for the formation of *β*-phase PVDF during the solution stage. The electrostatic interaction between the negatively charged surface of NP and the partially positively charged -CH dipoles causes the PVDF chains to be in all-trans conformation on the NP surface, which results in the formation of an electroactive *β*-crystalline phase. Therefore, the negatively charged surfaces of the NP are the sites of nucleation of the *β*-phase. Figure 2d represents the ion-dipole interaction of the PVDF chains with CuO NP schematically.

### 2.6. Characterization of CuO NPs and ES PVDF Composite NFs

The crystalline nature and functional group present in the prepared CuO NPs were studied and reported in our previous work [25]. Morphology of the NPs was analyzed using FESEM, (Thermo Fisher, FEI QUANTA 250 FEG Oregon, USA) and EDS mapping. The presence of crystalline phases and the *β*-crystallinity % in undoped and NPs-doped PVDF NFs were analyzed using XRD and FTIR. The morphological study of the ES neat PVDF and composite NFs was studied using FE-SEM after sputter coating with gold. The surface roughness of the ES NFs was analyzed using AFM, (Nanosurf QFM, Liestal, Switzerland) and the hydrophobic nature of the NFs was confirmed by WCA analysis studied with a sessile drop technique coupled with KRÜSS ADVANCE software, DSA25B. Output from the piezoelectric device, such as voltage (V) and current (μA), was measured using a digital Tektronix oscilloscope, (DPO4104, Tektronix, Oregon, USA). The sensitivity of the PENG device to body movements was studied using a BIOPAC MP-150 system with 100 MΩ input resistance and 0 dB gain. The data were saved in Acknowledge 4.2 software.

## 3. Results and Discussion

### 3.1. Surface Morphology and Elemental Analysis

**Figure 3:**
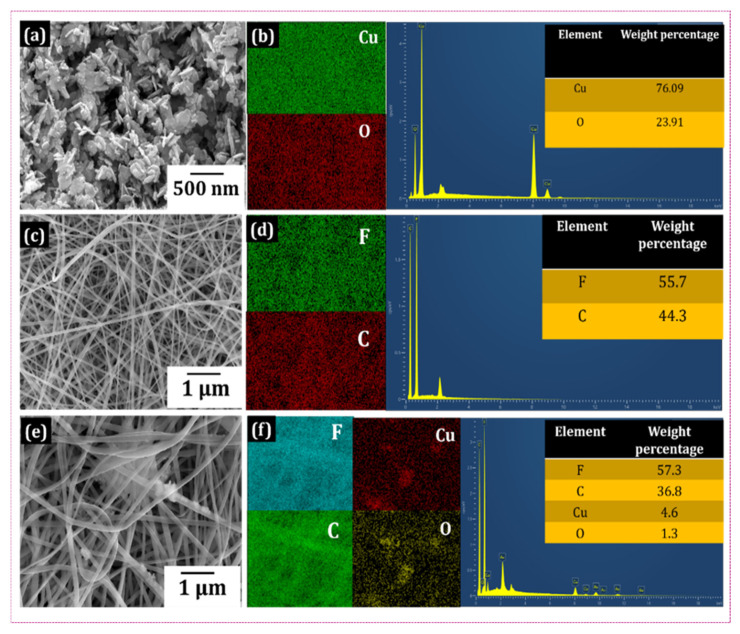
FE-SEM image and EDS analysis of (**a**,**b**) CuO NP; (**c**,**d**) 0PCu NF and (**e**,**f**) 5PCu NF, respectively.

The surface morphology of CuO NPs was studied using FE-SEM analysis. Figure 3a shows the surface morphology of the NPs with 500 nm magnification. The NPs have a uniform nanorod morphology. Figure 3b illustrates the NP’s elemental mapping and composition with the presence of Cu and O elements in 76% and 24%, respectively. This confirms the successful synthesis of CuO NPs. FE-SEM images of the 0PCu ES NF are shown in Figure 3c. The spinning conditions used in this experiment were ideal, as the 0PCu NF exhibited an even surface free of beads. Similarly, the CuO-PVDF composite NF 5PCu surface morphology displayed in Figure 3e confirms the incorporation of the nanofiller on the composite NF surface.

As the CuO content increases from 1–7 wt.-%, the fiber diameter decreases progressively, as further verified by ImageJ, 1.54r version, which calculated the diameters of 30 separate NFs and reported their average diameter. The values are shown in Table S1 of the Supporting Information (SI). The average fiber diameter of 0PCu is 365 nm, whereas the composite NFs of 1–7PCu decreased from 310 nm to 210 nm, aided by the polymer solution’s greater conductivity. Similarly, the standard deviation (SD) value decreased from 105 nm to 48 nm, which suggests that the NF’s size became more uniform. The EDS visuals of 0PCu and 5PCu NFs displayed in Figure 3d,f confirm the successful doping of CuO NP on the surface of PVDF NF [32]. The successful integration of CuO nanoparticles into PVDF fibers without detectable large-scale aggregation is confirmed by FESEM imaging and EDS elemental mapping. A higher-resolution cross-sectional study, however, might shed more light on dispersion at the nanoscale.

### 3.2. XRD Pattern

**Figure 4:**
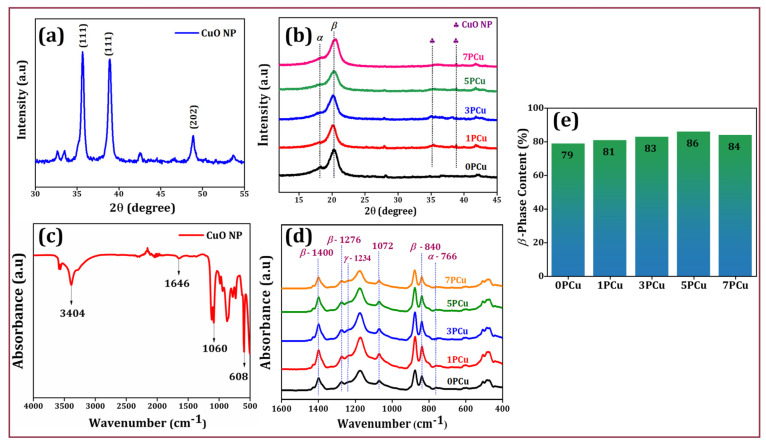
(**a**) XRD pattern of CuO NPs and (**b**) Neat PVDF and CuO-doped PVDF NFs; (**c**) FTIR analysis of CuO NP and (**d**) Neat PVDF and composite NFs; (**e**) Quantitative analysis of *β*-phase content (%).

As seen in Figure 4a, the CuO powder’s XRD pattern was examined throughout the 2θ range from 30° to 70°. The Miller indices of (002), (111), and (202) correspond to the identified peaks of CuO at 35.6°, 38.71°, and 48.67°, respectively, which match JCPDS no. 45-0937 [33]. The XRD peaks were used to compute the average crystallographic size using Scherrer’s equation (Equation (1)).

d = Kλ/(βCos θ)(1)

where θ represents the diffraction angle, β is the full width half maximum, K represents the form factor having an index of 0.9, and *λ* is the wavelength of the employed X-ray beam, which is approximately 1.54 Å. The mean crystallographic size of the CuO NP that matched the highest peak in the XRD was found to be 26 nm.

XRD analysis was performed on both neat and CuO-incorporated PVDF ES NFs (Figure 4b). In the case of neat (0PCu) NF, characteristic diffraction peaks corresponding to both the crystalline non-polar *α*- and polar *β*- phases were observed. For the composite NFs, additional diffraction peaks were seen due to the presence of CuO nanofiller, along with *α*- and *β*-phases of PVDF. A specific diffraction peak at 18.4° corresponding to the (020) plane signifies the *α*-phase. Similarly, the peak at 20.6° corresponds to the (200) plane, indicating the formation of the electroactive *β*-phase. Notably, the intensity of the *β*-phase peak increased with the inclusion of CuO, suggesting that the nanofiller plays a significant role in promoting the electroactive *β*-phase within the PVDF matrix.

### 3.3. FT-IR Studies

The FTIR characterization verified the presence of functional groups within the CuO NPs (Figure 4c). The Cu-O vibrational deformation was exhibited by a peak at 608 cm^−1^. The absorption peak at 1646 cm^−1^ indicates both symmetric and asymmetric stretching of the Cu-O bond. The C-O plane was attributed to the 1060 peak. Figure 4d displays FTIR spectra of the prepared NFs. The absorption peaks at 840 cm^−1^, 1276 cm^−1^, and 1400 cm^−1^ correspond to the polar β-phase of PVDF, whereas the peak at 766 cm^−1^ corresponds to the *α*-phase. In the case of the 0PCu NF, the *α*-phase peak is distinctly visible; however, this peak gradually weakens in the composite NFs as the CuO content increases. To quantify the *β*-phase content in the PVDF NF, the Lambert–Beer law (refer to Equation (2)) was employed [34], utilizing the absorbance intensities at 766 cm^−1^ and 840 cm^−1^.

F(*β*) = [(*A_β_*)/((1.26 × *A_α_*) + *A_β_*)] × 100 (2)

In this analysis, Aα and Aβ represent the absorbance values at 766 cm^−1^ and 840 cm^−1^, respectively. The β-phase content in 0PCu NF was 79%. For the composite NFs, the β-phase increased to 81%, 83%, 86%, and 84% for CuO concentrations of 1, 3, 5, and 7 wt.-%, respectively (Figure 4e). As the CuO content increased from 0 to 5 wt.-%, the proportion of the β-phase also rose from 79% to 86%, as detailed in Table S2 in SI. This increase is due to the strong interaction between the negatively charged surface of CuO and the positively charged CH_2_ dipoles of PVDF chains, which promotes alignment of the PVDF chains in an all-trans conformation [35]. During electrospinning, molecular chain alignment along the fiber axis is induced by applying a high electric field (typically in the kV range) and rapid jet stretching. This process converts the non-polar α-phase into the electroactive β-phase [36]. A self-poling effect may also occur due to the strong electric field, which orients the dipoles without needing additional post-poling treatment. Previous studies have documented a similar increase in the β-phase caused by electrospinning [37].

### 3.4. WCA Measurement

**Figure 5:**
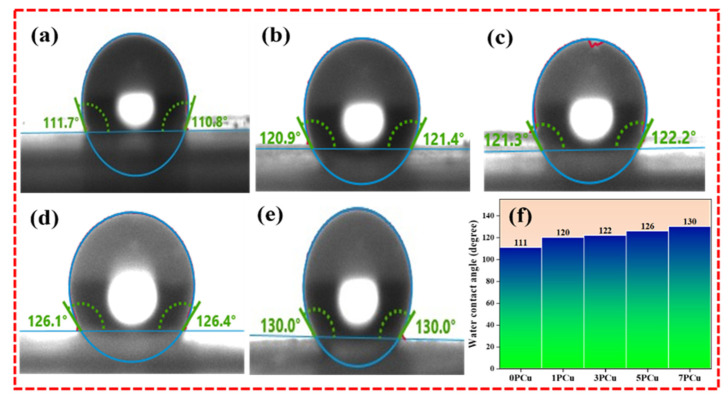
WCA of (**a**) 0PCu; (**b**) 1PCu; (**c**) 3PCu; (**d**) 5PCu; (**e**) 7PCu; (**f**) The quantitative analysis of WCA.

Materials with hydrophilic surfaces tend to absorb moisture from the surrounding air, forming a thin layer of water molecules on the fiber surface that can influence their electrical behavior. To evaluate the hydrophilic or hydrophobic characteristics of both undoped PVDF and CuO-doped NFs, WCA measurements were conducted. NF samples were cut into 1 cm × 1 cm pieces, mounted on glass slides, and a 2 mL droplet of deionized water was placed on their surfaces. The WCA was recorded at 10-s intervals, and the results for both neat and composite NFs are illustrated in Figure 5a–e. The contact angle values observed for 0PCu, 1PCu, 3PCu, 5PCu and 7PCu were 111°, 120°, 122°, 126° and 130°, respectively. Figure 5f presents the quantitative comparison of WCA values. The increasing WCA upon addition of CuO NPs indicates an enhancement in the hydrophobic nature of the PVDF matrix. This improved hydrophobicity makes the NFs suitable for applications in humid conditions. According to the Wenzel model, the elevated WCA in the 7PCu sample can be attributed to its increased surface roughness, which enhances hydrophobicity [22,38]. The wettability of the NF is crucial for practical applications, and WCA analysis confirms that a hydrophobic surface is more desirable.

### 3.5. AFM Analysis

Using 2D and 3D AFM images, the surface roughness of neat PVDF and PVDF/CuO composite NFs was analyzed and compared (Figure S1 in SI). The average roughness (*R*_a_) and root mean square roughness (*R*_q_) values for both neat PVDF and the PVDF/CuO composites are detailed in Table S2 in the SI. The measured *R*_a_ values were 170, 510, 824, 1161, and 568 nm for 0PCu, 1PCu, 3PCu, 5PCu, and 7PCu, respectively. It was observed that increasing the NPs content in the PVDF matrix led to a progressive rise in surface roughness. Among all the samples, 5PCu displayed the highest *R*_a_ value. AFM analyses showed that the PVDF nanofibers’ surface roughness changed when CuO nanoparticles were added. Surface morphology was comparatively smoother in pristine PVDF samples, but surface roughness increased in CuO-loaded samples. To better understand this behavior, measurements of the water contact angle were linked to roughness values [39]. CuO nanoparticles and the PVDF matrix appear to interact at the interface, as evidenced by the significant shift in contact angle observed following CuO incorporation. PVDF is naturally hydrophobic. Nanoparticle inclusion and altered surface properties resulting from the CuO–PVDF interaction may thus be the cause of the enhanced roughness. Surface roughness is not thought to be a major determinant of piezoelectric output.

### 3.6. Electrical Measurements

Using a custom-built setup, the output peak-to-peak voltage (*V*_p-p_) of both neat and composite PVDF-based PENG was measured at a repeated compression frequency of 1 Hz and an applied force of 9.81 N (equal to 1 kgf). With an active area of 04 cm^2^, the equivalent compressive stress was around 2.4525 kPa (Force/Area). Small changes in the thickness of the electrospun fiber material can have an impact on the reported output voltage because the active layer’s thickness affects the generated piezoelectric potential [40]. The peak-to-peak output voltage was normalized to the measured fiber thickness to provide a fair evaluation of the intrinsic electromechanical performance of the various compositions. Equation (3) was utilized to compute the normalized voltage.

Normalized voltage (*V*_p-p_(normalized)) = (*V*_p-p_/Fiber thickness in µm) × 100 (3)

In this case, the multiplying factor of 100 is only used as a scaling constant to display the normalized results in a comfortable numerical range; it does not affect the comparison trends between samples. A digital micrometer was used to measure the thickness of each electrospun fiber material at five separate points. To reduce measurement uncertainty, the average value was utilized in normalization. The normalized voltage and related thickness values for each sample are provided in Table 1.

**Figure 6:**
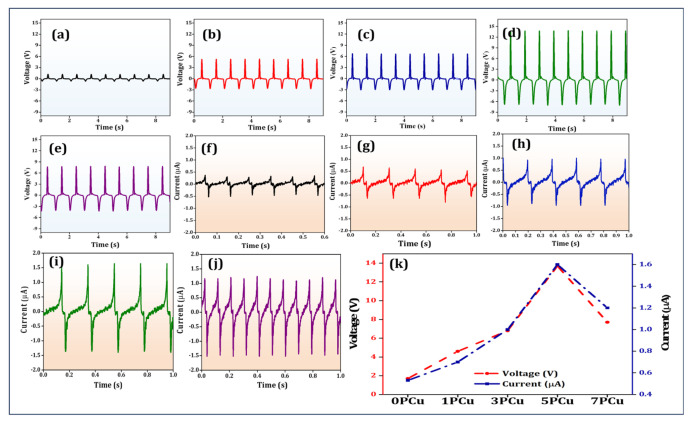
(**a**–**e**) Piezoelectric output voltage. (**f**–**j**) Output current of 0PCu, 1PCu, 3PCu, 5PCu, and 7PCu, respectively. (**k**) Quantitative analysis of output voltage and current of the PENG devices.

The sensor made from 0PCu produced an output of 1.7 V, whereas the composite PENG devices exhibited higher output voltages (Figure 6a–e)—1PCu: 4.2 V, 3PCu: 6.8 V, 5PCu: 13.7 V, and 7PCu: 8.1 V. Among all the samples, the 5PCu composite demonstrated approximately 8 times higher piezoelectric voltage output than the 0PCu sample. The enhanced yield at 5 wt.% CuO is likely due to an effective combination of higher β-phase concentration, optimized fiber shape, and enhanced interfacial contacts. The nonlinear performance pattern shows that excessive filler loading leads to competing effects, including aggregation and structural variability. Figure 6f–j depicts the short-circuit current (*I*_sc_)—0PCu: 0.53 μA, 1PCu: 0.7 μA, 3PCu: 1.0 μA, 5PCu: 1.6 μA and 7PCu: 1.2 μA. These results indicate enhanced electrical performance upon the incorporation of NPs into the PVDF matrix [41]. The quantitative voltage and current outputs of the tested devices are illustrated in Figure 6k. This indicates that the 5PCu-based PENG device has strong potential for energy-harvesting applications. Further, we compare our data with PENG sensors developed by other researchers, showing superior electrical performance of our PCu composite PENG (See Table 2).

Equation (4) was used to estimate the maximal instantaneous power output.

P = V × I(4)

The device achieved a peak voltage of 13.7 V and current of 1.6 µA under repeated mechanical load, resulting in an absolute power of roughly 21.9 µW.

Lastly, 14,000 cycles of continuous operation at a fixed frequency of 1 Hz and a load of 9.81 N were used to evaluate the mechanical stability and effectiveness of the 5PCu PENG device. The findings are displayed in Figure S2 in the SI. Every 2000 cycles, the output was continuously assessed. Even after constant use, no changes were seen. As a result, the 5PCu PENG demonstrated exceptional electrical stability and is suitable for real-time energy-harvesting systems.

### 3.7. Applications of 5PCu-Based PENG Device

#### 3.7.1. Wearable Applications

**Figure 7:**
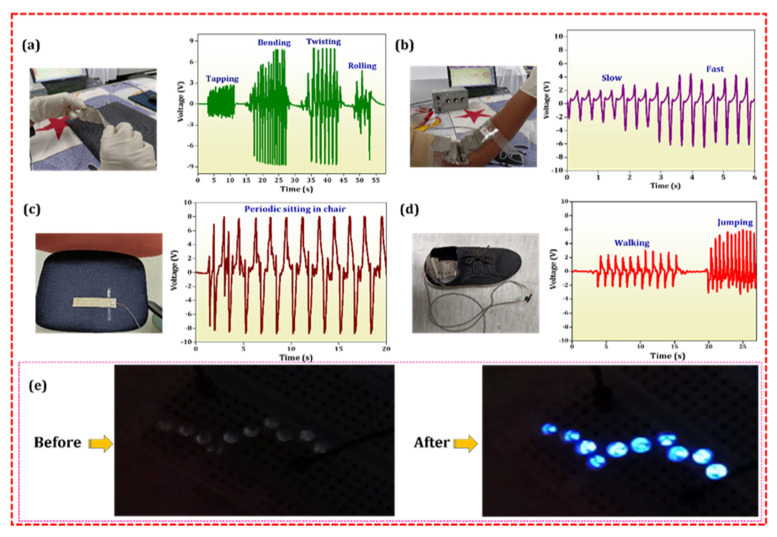
Real-time wearable applications of the 5PCu PENG device. (**a**) Tapping. (**b**) Elbow movement. (**c**) Periodic sitting. (**d**) Walking and jumping. (**e**) Optical images of 10 LEDs before and after connecting the PENG device.

The optimized 5PCu PENG device output was evaluated using a 100 MΩ load resistance and 0 dB input impedance. As depicted in Figure 7a, voltage outputs recorded for different body motions were 4.2 V for tapping, 16.2 V for bending, 16.4 V for twisting, and 12.5 V for rolling (refer to Video S1 in SI). Among these, twisting elicited the highest voltage, indicating the sensor’s strong responsiveness to such deformation. The sensor was fixed near the joint to study elbow movement, and voltage variations were observed during periodic arm stretches. Figure 7b shows that when the arm’s movement angle increased from 30° to 180°, the sensor’s voltage rose from 6.4 V to 10.8 V. Additionally, an output of 16.2 V was recorded while the subject was seated (Figure 7c). For motion detection during walking and running, the sensor was attached to the shoe sole, producing voltages of 5.2 V and 9.1 V, respectively (Figure 7d). While walking or jumping, the applied force is roughly 7–12 N, and during moderate finger tapping, it is 1–5 N [50,51]. Similarly, the estimated motion frequency will be between ~1 and 3 Hz. Although these studies lacked accurate calibration of force and frequency, the applied forces fall within the usual range of human motion described in the literature. These results highlight the potential of the developed 5PCu PENG device for broader biomedical applications, particularly in assisting with hand function recovery and gait training following a stroke or traumatic brain injury.

#### 3.7.2. Energy-Harvesting Application

To evaluate the real-time energy-harvesting capabilities of the PENG device, experiments were conducted using a 1 kgf force at 1 Hz to power light-emitting diodes (LEDs). As shown in Figure 7e, the 5PCu-based PENG successfully illuminated 10 LEDs simultaneously. The device was connected to the LEDs through a bridge rectifier, demonstrating its ability to directly power electronic components. The operation of the LEDs is shown in Video S2 in the SI.

## 4. Conclusions

This work reports the fabrication of CuO nanoparticle-loaded PVDF electrospun nanofibers with filler concentrations varying from 0 to 7 wt.-% for application in wearable PENGs. The electrospinning process produced continuous nanofibers, and microscopic examination verified the effective incorporation of CuO within the PVDF fiber network without disrupting fiber formation. Structural investigations using FTIR and XRD confirmed that the presence of CuO enhances the electroactive *β*-phase in PVDF. The *β*-phase content increased from 79% in pristine PVDF to 86% at an optimal CuO loading of 5 wt.-%, indicating improved phase transformation due to nanoparticle inclusion.

The electrical characterization results revealed that the device containing 5 wt.-% CuO delivered the highest performance among all compositions. When subjected to a periodic mechanical load of 1.0 kgf at 1.0 Hz, the optimized nanogenerator produced a peak-to-peak voltage of 13.7 V, nearly eight times that of the undoped PVDF device (1.7 V). A corresponding improvement in short-circuit current was also observed, increasing from 0.7 μA to 1.6 μA. Increasing the CuO concentration beyond 5 wt.-% led to a decline in output, suggesting that excessive filler content may adversely affect electromechanical efficiency.

The developed nanogenerator was further evaluated under various human motion conditions, including walking, running, tapping, jumping, and elbow bending, demonstrating its ability to operate as a self-powered wearable sensor. The improvement in electrical output achieved through optimized nanoparticle incorporation helps address the low-output limitations commonly associated with PVDF-based piezoelectric devices. These findings indicate that the PVDF/CuO electrospun nanofiber system has significant potential for wearable energy-harvesting and motion-sensing applications.

## Figures and Tables

**Table 1:** Relation between normalized peak-to-peak voltage and fiber thickness.

Fiber	*V*_p-p_ (V)	Fiber Thickness (µm)	*V*_p-p_ (Normalized) (V)
0PCu	1.6	94	1.7
1PCu	3.5	83	4.2
3PCu	5.9	86	6.8
5PCu	11.4	83	13.7
7PCu	7.7	96	8.1

**Table 2:** Performances of electrospun PVDF-based PENGs.

S. No.	Additive	Output	Ref.
1	NiFe_2_O_4_	Voltage 5 V	[42]
2	FeCl_3_ and CsPbBr_3_	Voltage 10.3 V, Current density 1.29 µA/cm^2^	[43]
3	BiCl_3_	Voltage 1.1 V Current 2 µA	[44]
4	Graphene	Voltage 7.9 V	[45]
5	MWCNT (0.008)	Voltage 0.62 V	[21]
6	HNT-PANi	Voltage 7.2 V, Current 0.75 μA	[46]
7	ZnO	Voltage ~480 mV	[24]
8	Zn(Ac)_2_	Voltage 0.359 V	[47]
9	Nanodiamonds (1.0)	Voltage 6.0 V	[48]
10	FAPbBr_3_ (12)	Voltage 8 V	[49]
11	CuO	Voltage 13.7 V Current 1.6 µA	This Work

## Data Availability

The raw data supporting the conclusions of this article will be made available by the authors on request.

## Supplementary Materials

The following supporting information can be downloaded at https://www.mdpi.com/article/10.3390/polym18060699/s1. Table S1: Fiber diameter distribution of neat and composite PVDF nanofibers; Table S2: β-phase % and 2-D AFM analysis data of neat and composite PVDF NFs; Figure S1: High-resolution (a–e) 2-D and (a’–e’) 3-D AFM images of 0PCu, 1PCu, 3PCu, 5PCu, and 7PCu NFs. Figure S2: Mechanical stability of 5PCu-PENG for 14,000 cycles. Video S1: Wearable sensor demonstration of 5PCu-PENG under various human motions. Video S2: Illumination of 10 LEDs using 5PCu-PENG.
