# Supplementary material for: Eco-Friendly Copper Oxide Nanoparticles Incorporated Polyvinylidene Difluoride Electrospun Nanofibers as Wearable Piezoelectric Nanogenerator
Source: Polymers (Basel). 2026 Mar 13;18(6):699. doi: 10.3390/polym18060699 (PMC13030057; doi:10.3390/polym18060699)
Supplement: Supplementary file 1 [file polymers-18-00699-s001.zip › Supporting Information (modified according to proof correction).pdf]

Supporting Information

Eco-Friendly Copper Oxide Nanoparticles Incorporated Polyvinylidene Difluoride Electrospun Nanofibers as Wearable Piezoelectric Nanogenerator

Amrutha Bindhu [1,†], Sathiyanathan Ponnan [2,†], Shamim Reza Mohammad [3], Riya Karmakar [4], Hongdoo Kim [3], Arvind Mukundan [4,5,*] and Anand Prabu Arun [1,*]

[1] Department of Chemistry, School of Advanced Sciences, Vellore Institute of Technology, Vellore 632014, India; amrutha.b2020@vitstudent.ac.in
[2] Department of Chemistry, Vel Tech Rangarajan Dr. Sagunthala R&D Institute of Science and Technology, Chennai 600062, India; sat.bni@gmail.com
[3] Department of Materials Science and Engineering, College of Engineering, Kyung Hee University, Yongin-si 17104, Gyeonggi-do, Republic of Korea; reza13@khu.ac.kr (S.R.M.); hdkim@khu.ac.kr (H.K.)
[4] School of Engineering and Technology, Sanjivani University, Sanjivani Factory, Kopargaon 423603, India; karmakarriya345@gmail.com
[5] Department of Biomedical Imaging, Chennai Institute of Technology, Chennai 600069, India
* Correspondence: arvindmukund96@gmail.com (A.M.); anandprabu@vit.ac.in (A.P.A.)
† These authors contributed equally to this work.

Table S1: Fiber diameter distribution of neat and composite PVDF NFs.

Sample Code	Distribution of fiber diameter (nm)			
	Mean	Standard deviation	Min	Max
0PCu	365	105	179	689
1PCu	310	86	144	666
3PCu	300	80	184	540
5PCu	289	72	162	454
7PCu	210	48	139	386

Table S2: β-phase % and 2-D AFM analysis data of neat and composite PVDF NFs.

Sample Code	Absorption Intensity		F(β)%	R_a (nm)	R_q (nm)
	A_α	A_β			
0PCu	0.0311	0.1545	79	170.0	215.7
1PCu	0.0430	0.239	81	431.0	542.0
3PCu	0.0291	0.1785	83	510.0	638.0
5PCu	0.0261	0.2111	86	1161.0	1499.0
7PCu	0.0193	0.1279	84	568.0	768.0

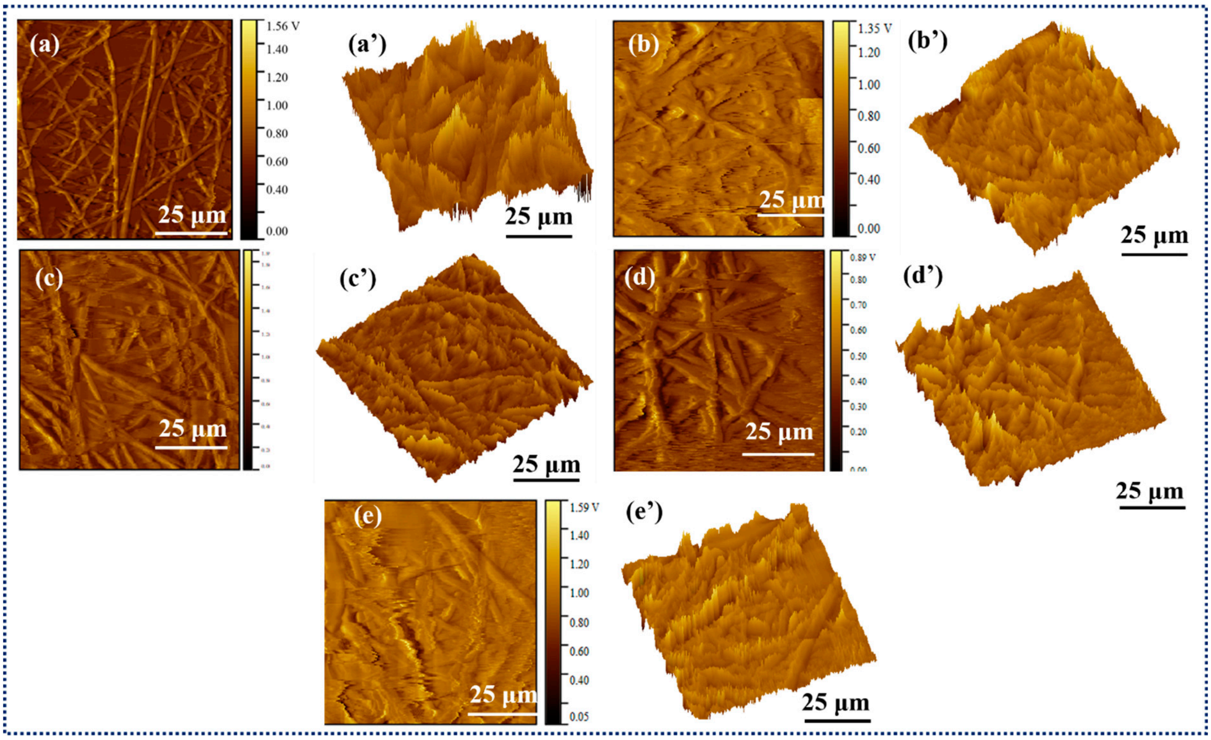

Figure S1. High-resolution **(a- e)** 2-D and **(a′ -e′)** 3-D AFM images of 0PCu, 1PCu, 3PCu, 5PCu and 7PCu NFs.

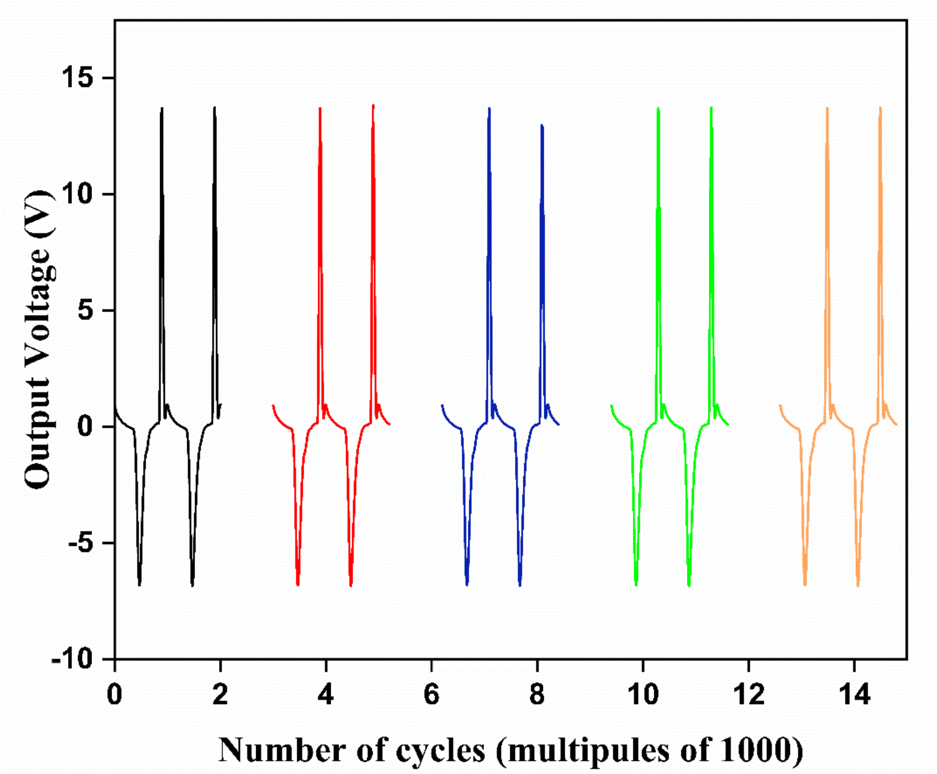

Figure S2. Mechanical stability of 5PCu-PENG for 14000 cycles.
